# Supplementary material for: Mitochondrial DNA Changes in Respiratory Complex I Genes in Brain Gliomas
Source: Biomedicines. 2023 Apr 15;11(4):1183. doi: 10.3390/biomedicines11041183 (PMC10135735; doi:10.3390/biomedicines11041183)
Supplement: Supplementary file 1 [file biomedicines-11-01183-s001.zip › biomedicines-2275831-supplementary.pdf]

Supplementary Materials

Mitochondrial DNA Changes in Respiratory Complex I Genes in Brain Gliomas

Paulina Kozakiewicz, Ludmiła Grzybowska-Szatkowska, Marzanna Ciesielka, Paulina Całka, Jacek Osuchowski, Paweł Szmygin, Bożena Jarosz, Marta Ostrowska-Leśko, Jarosław Dudka, Angelika Tkaczyk-Wlizło and Brygida Ślaska

ND1 evaluation with TMHMM Server

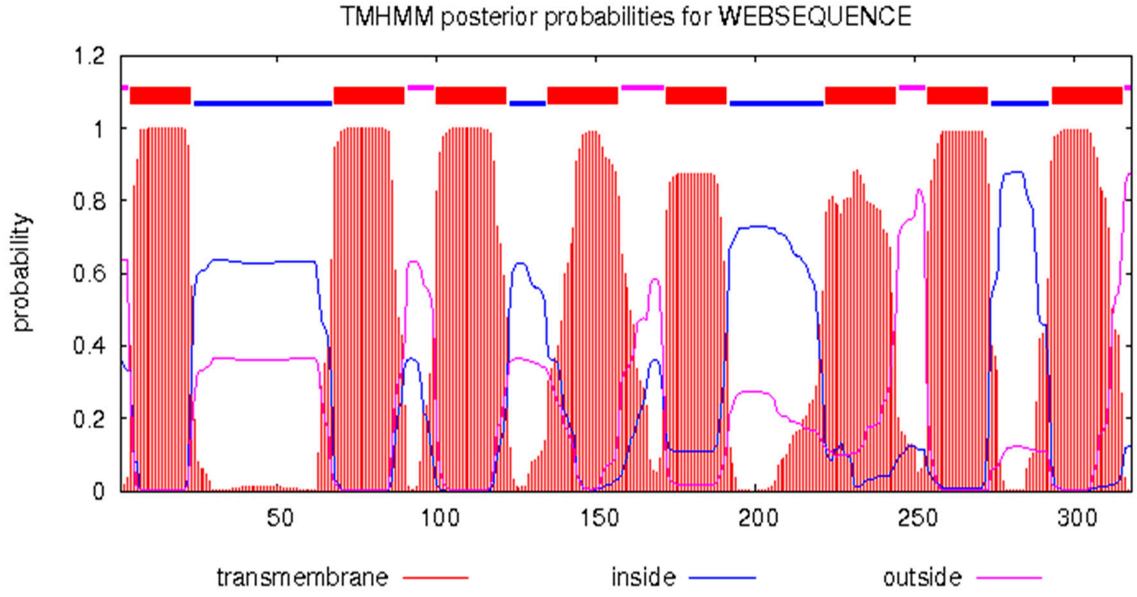


```
# WEBSEQUENCE Length: 318
# WEBSEQUENCE Number of predicted TMHs:  8
# WEBSEQUENCE Exp number of AAs in TMHs: 167.91966
# WEBSEQUENCE Exp number, first 60 AAs:  19.37957
# WEBSEQUENCE Total prob of N-in:        0.36409
# WEBSEQUENCE POSSIBLE N-term signal sequence
WEBSEQUENCE    TMHMM2.0      outside      1     3
WEBSEQUENCE    TMHMM2.0      TMhelix      4    23
WEBSEQUENCE    TMHMM2.0      inside      24    67
WEBSEQUENCE    TMHMM2.0      TMhelix     68    90
WEBSEQUENCE    TMHMM2.0      outside     91    99
WEBSEQUENCE    TMHMM2.0      TMhelix    100   122
WEBSEQUENCE    TMHMM2.0      inside     123   134
WEBSEQUENCE    TMHMM2.0      TMhelix    135   157
WEBSEQUENCE    TMHMM2.0      outside    158   171
WEBSEQUENCE    TMHMM2.0      TMhelix    172   191
WEBSEQUENCE    TMHMM2.0      inside     192   221
WEBSEQUENCE    TMHMM2.0      TMhelix    222   244
WEBSEQUENCE    TMHMM2.0      outside    245   253
WEBSEQUENCE    TMHMM2.0      TMhelix    254   273
WEBSEQUENCE    TMHMM2.0      inside     274   292
WEBSEQUENCE    TMHMM2.0      TMhelix    293   315
WEBSEQUENCE    TMHMM2.0      outside    316   318
```

Figure S1. 1 Reference protein ND1 according to the Cambridge sequence (red font indicates the places where the amino acid shift took place).

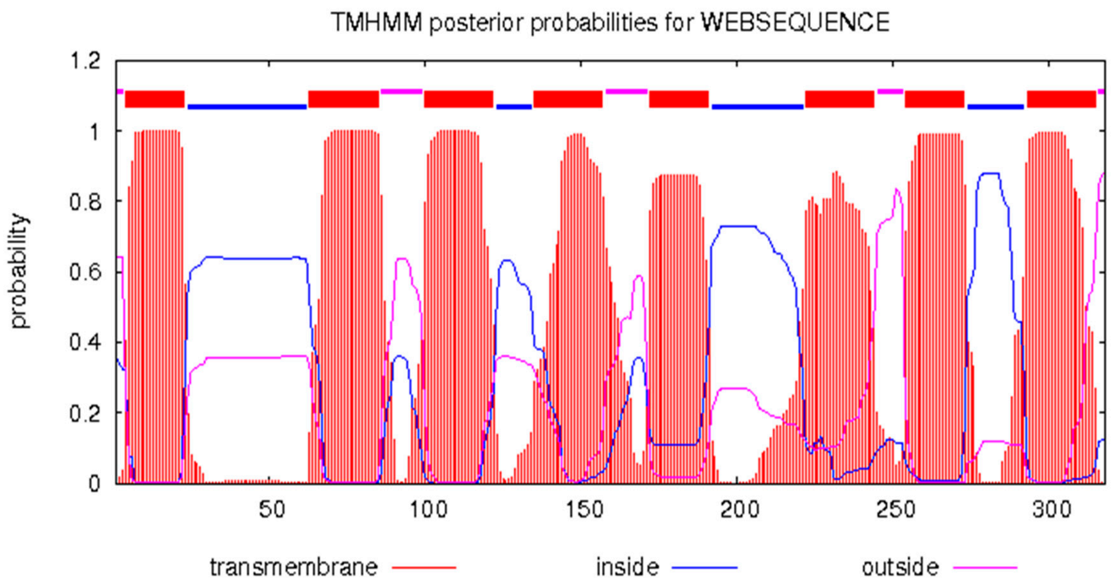


```
# WEBSEQUENCE Length: 318
# WEBSEQUENCE Number of predicted TMHs:  8
# WEBSEQUENCE Exp number of AAs in TMHs: 168.01817
# WEBSEQUENCE Exp number, first 60 AAs:  19.33487
# WEBSEQUENCE Total prob of N-in:        0.35825
# WEBSEQUENCE POSSIBLE N-term signal sequence
WEBSEQUENCE     TMHMM2.0        outside       1     3
WEBSEQUENCE     TMHMM2.0        TMhelix       4    23
WEBSEQUENCE     TMHMM2.0        inside       24    62
WEBSEQUENCE     TMHMM2.0        TMhelix      63    85
WEBSEQUENCE     TMHMM2.0        outside      86    99
WEBSEQUENCE     TMHMM2.0        TMhelix     100   122
WEBSEQUENCE     TMHMM2.0        inside      123   134
WEBSEQUENCE     TMHMM2.0        TMhelix     135   157
WEBSEQUENCE     TMHMM2.0        outside     158   171
WEBSEQUENCE     TMHMM2.0        TMhelix     172   191
WEBSEQUENCE     TMHMM2.0        inside      192   221
WEBSEQUENCE     TMHMM2.0        TMhelix     222   244
WEBSEQUENCE     TMHMM2.0        outside     245   253
WEBSEQUENCE     TMHMM2.0        TMhelix     254   273
WEBSEQUENCE     TMHMM2.0        inside      274   292
WEBSEQUENCE     TMHMM2.0        TMhelix     293   315
WEBSEQUENCE     TMHMM2.0        outside     316   318
```

Figure S1. 2 The protein ND1 with change T67A (red font indicates the shift of aminoacids in the protein).

ND3 evaluation with TMHMM Server

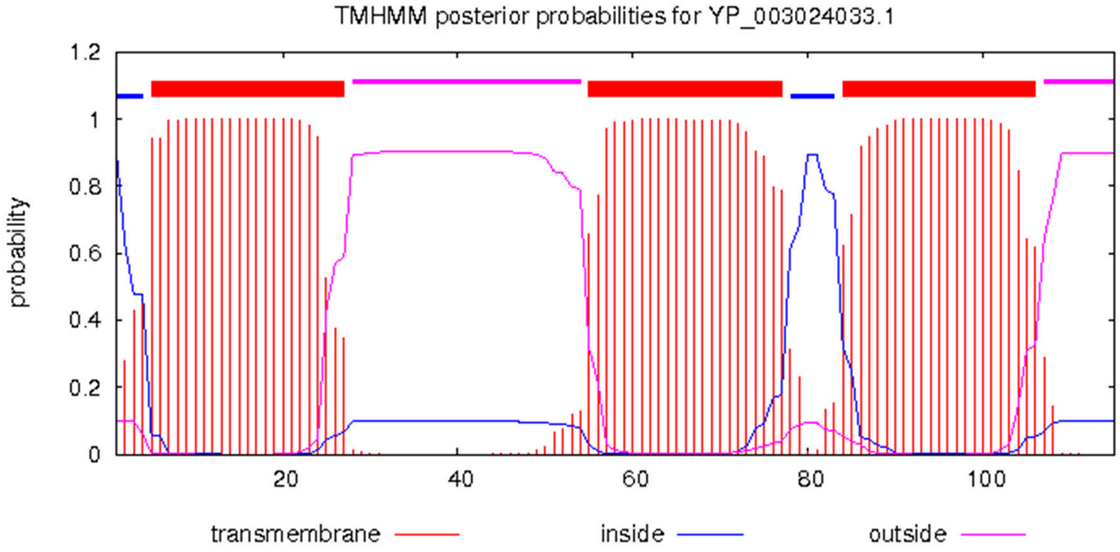


```
# WEBSEQUENCE Length: 115
# WEBSEQUENCE Number of predicted TMHs:  3
# WEBSEQUENCE Exp number of AAs in TMHs: 66.83257
# WEBSEQUENCE Exp number, first 60 AAs:  28.03563
# WEBSEQUENCE Total prob of N-in:        0.90203
# WEBSEQUENCE POSSIBLE N-term signal sequence
WEBSEQUENCE     TMHMM2.0        inside        1     4
WEBSEQUENCE     TMHMM2.0        TMhelix       5    27
WEBSEQUENCE     TMHMM2.0        outside      28    54
WEBSEQUENCE     TMHMM2.0        TMhelix      55    77
WEBSEQUENCE     TMHMM2.0        inside       78    83
WEBSEQUENCE     TMHMM2.0        TMhelix      84   106
WEBSEQUENCE     TMHMM2.0        outside     107   115
```

Figure S2. 1 Reference protein ND3 according to the Cambridge sequence (red font indicates the places where the amino acid shift took place).

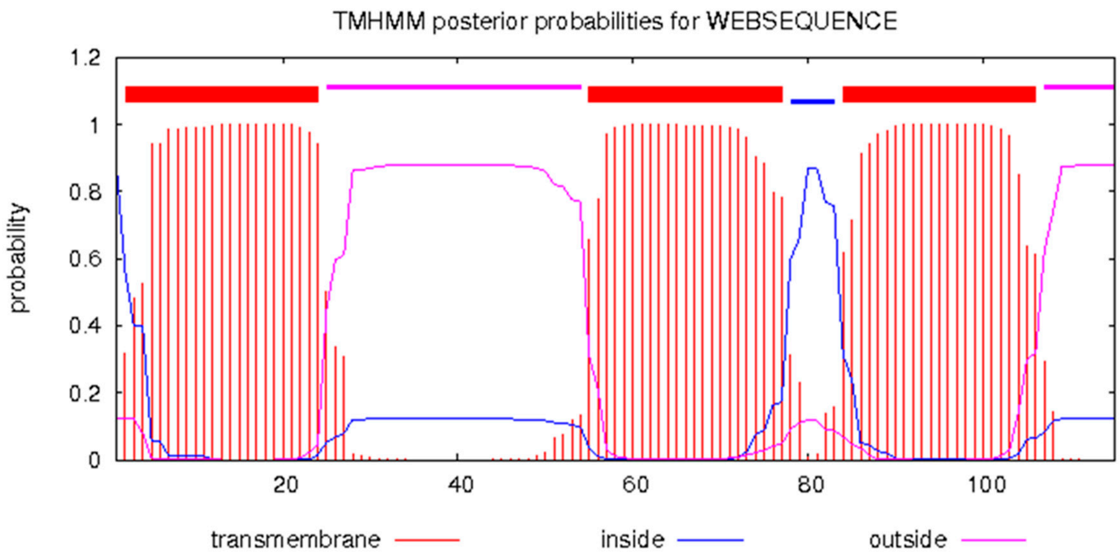


```
# WEBSEQUENCE Length: 115
# WEBSEQUENCE Number of predicted TMHs:  3
# WEBSEQUENCE Exp number of AAs in TMHs: 66.82263
# WEBSEQUENCE Exp number, first 60 AAs:  28.06473
# WEBSEQUENCE Total prob of N-in:        0.87812
# WEBSEQUENCE POSSIBLE N-term signal sequence
WEBSEQUENCE     TMHMM2.0        inside       1     1
WEBSEQUENCE     TMHMM2.0        TMhelix      2    24
WEBSEQUENCE     TMHMM2.0        outside     25    54
WEBSEQUENCE     TMHMM2.0        TMhelix     55    77
WEBSEQUENCE     TMHMM2.0        inside      78    83
WEBSEQUENCE     TMHMM2.0        TMhelix     84   106
WEBSEQUENCE     TMHMM2.0        outside    107   115
```

Figure S2. 2 Test protein ND3 with change I9T (red font indicates the shift of amino acids in the protein).

ND5 evaluation with TMHMM Server

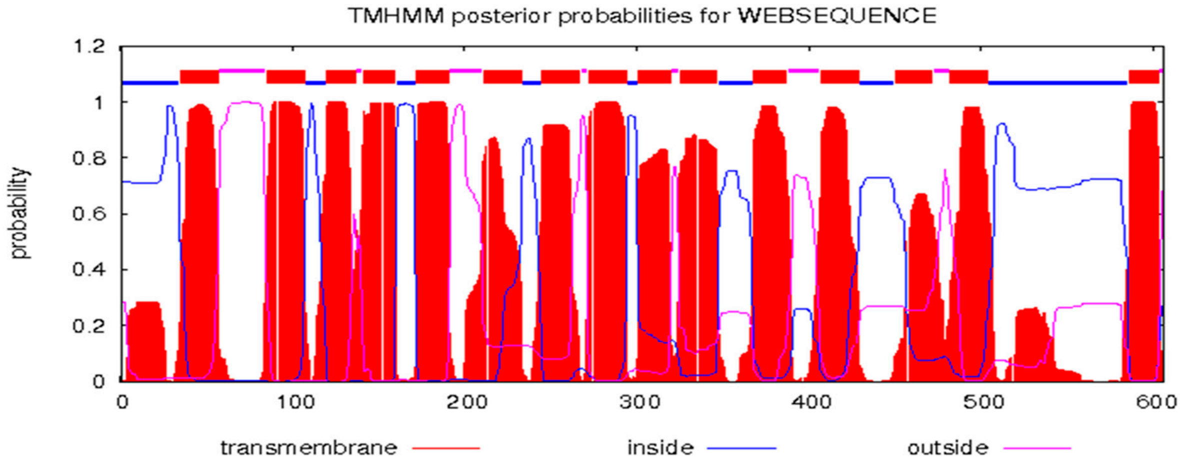


```
# WEBSEQUENCE Length: 606
# WEBSEQUENCE Number of predicted TMHs:  15
# WEBSEQUENCE Exp number of AAs in TMHs: 310.80792
# WEBSEQUENCE Exp number, first 60 AAs:  26.92284
# WEBSEQUENCE Total prob of N-in:        0.71498
# WEBSEQUENCE POSSIBLE N-term signal sequence
WEBSEQUENCE     TMHMM2.0        inside       1    34
WEBSEQUENCE     TMHMM2.0        TMhelix     35    57
WEBSEQUENCE     TMHMM2.0        outside     58    84
WEBSEQUENCE     TMHMM2.0        TMhelix     85   107
WEBSEQUENCE     TMHMM2.0        inside     108   119
WEBSEQUENCE     TMHMM2.0        TMhelix    120   137
WEBSEQUENCE     TMHMM2.0        outside    138   140
WEBSEQUENCE     TMHMM2.0        TMhelix    141   160
WEBSEQUENCE     TMHMM2.0        inside     161   171
WEBSEQUENCE     TMHMM2.0        TMhelix    172   191
WEBSEQUENCE     TMHMM2.0        outside    192   210
WEBSEQUENCE     TMHMM2.0        TMhelix    211   233
WEBSEQUENCE     TMHMM2.0        inside     234   244
WEBSEQUENCE     TMHMM2.0        TMhelix    245   267
WEBSEQUENCE     TMHMM2.0        outside    268   271
WEBSEQUENCE     TMHMM2.0        TMhelix    272   294
WEBSEQUENCE     TMHMM2.0        inside     295   300
WEBSEQUENCE     TMHMM2.0        TMhelix    301   320
WEBSEQUENCE     TMHMM2.0        outside    321   324
WEBSEQUENCE     TMHMM2.0        TMhelix    325   347
WEBSEQUENCE     TMHMM2.0        inside     348   367
WEBSEQUENCE     TMHMM2.0        TMhelix    368   387
WEBSEQUENCE     TMHMM2.0        outside    388   406
WEBSEQUENCE     TMHMM2.0        TMhelix    407   429
WEBSEQUENCE     TMHMM2.0        inside     430   449
WEBSEQUENCE     TMHMM2.0        TMhelix    450   472
WEBSEQUENCE     TMHMM2.0        outside    473   481
WEBSEQUENCE     TMHMM2.0        TMhelix    482   504
WEBSEQUENCE     TMHMM2.0        inside     505   585
WEBSEQUENCE     TMHMM2.0        TMhelix    586   603
WEBSEQUENCE     TMHMM2.0        outside    604   606
```

Figure S3. 1 ND5 reference protein according to the Cambridge sequence.

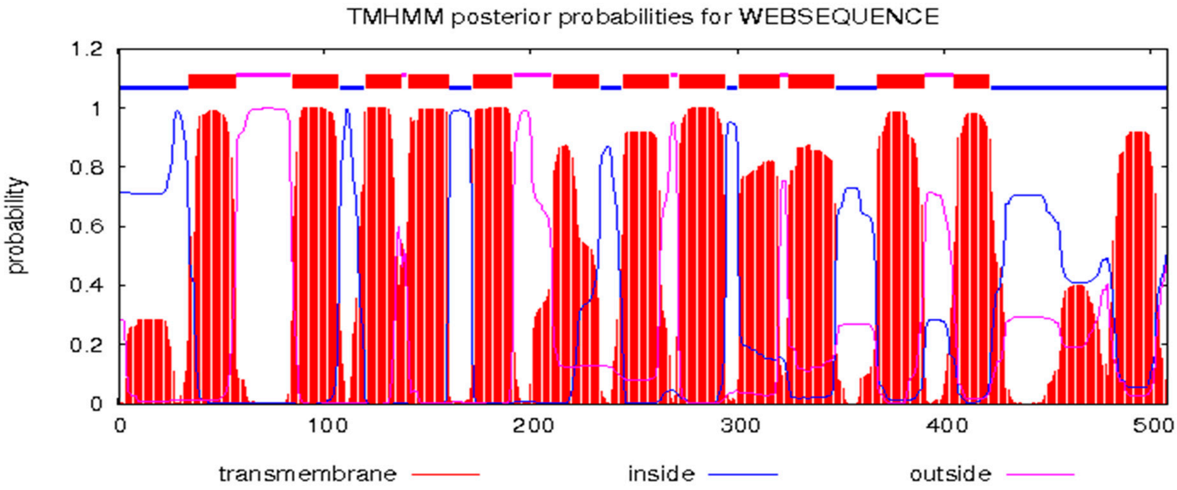


```
# WEBSEQUENCE Length: 508
# WEBSEQUENCE Number of predicted TMHs:  12
# WEBSEQUENCE Exp number of AAs in TMHs: 277.15301
# WEBSEQUENCE Exp number, first 60 AAs:  26.92284
# WEBSEQUENCE Total prob of N-in:        0.71498
# WEBSEQUENCE POSSIBLE N-term signal sequence
WEBSEQUENCE    TMHMM2.0       inside       1    34
WEBSEQUENCE    TMHMM2.0       TMhelix     35    57
WEBSEQUENCE    TMHMM2.0       outside     58    84
WEBSEQUENCE    TMHMM2.0       TMhelix     85   107
WEBSEQUENCE    TMHMM2.0       inside     108   119
WEBSEQUENCE    TMHMM2.0       TMhelix    120   137
WEBSEQUENCE    TMHMM2.0       outside    138   140
WEBSEQUENCE    TMHMM2.0       TMhelix    141   160
WEBSEQUENCE    TMHMM2.0       inside     161   171
WEBSEQUENCE    TMHMM2.0       TMhelix    172   191
WEBSEQUENCE    TMHMM2.0       outside    192   210
WEBSEQUENCE    TMHMM2.0       TMhelix    211   233
WEBSEQUENCE    TMHMM2.0       inside     234   244
WEBSEQUENCE    TMHMM2.0       TMhelix    245   267
WEBSEQUENCE    TMHMM2.0       outside    268   271
WEBSEQUENCE    TMHMM2.0       TMhelix    272   294
WEBSEQUENCE    TMHMM2.0       inside     295   300
WEBSEQUENCE    TMHMM2.0       TMhelix    301   320
WEBSEQUENCE    TMHMM2.0       outside    321   324
WEBSEQUENCE    TMHMM2.0       TMhelix    325   347
WEBSEQUENCE    TMHMM2.0       inside     348   367
WEBSEQUENCE    TMHMM2.0       TMhelix    368   390
WEBSEQUENCE    TMHMM2.0       outside    391   404
WEBSEQUENCE    TMHMM2.0       TMhelix    405   422
WEBSEQUENCE    TMHMM2.0       inside     423   508
```

Figure S3. 2 The protein ND5 with mutation Y506STP.

Table S1. Biochemical values of complex I subunits depending on the presence of aminoacid residues in a given position of the protein.

Change in protein position	Theoretical isoelectric point	Aliphatic index	Instability index	Grand average of hydropathicity (GRAVY)	The helix percentage	Position in protein structure
Polymorphisms						
ND1						
Y304H (T4216C)	6.29	123.08	42.69 protein unstable	0.676	H13 = 4.86 alpha helix	transmembrane section
T67A (A3505G)	6.11	123.40	41.94 protein unstable	0.690	H5 = 0.07 alpha helix	mitochondrial matrix, low complexity area
T229M (C3992T)	6.11	123.08	44.06 protein unstable	0.690	H10 = 2.37 alpha helix	transmembrane section, low complexity area
T240A (A4024G)	6.11	123.40	41.94 protein unstable	0.690	H10 = 0.79 alpha helix	transmembrane section, low complexity area
T263A (A4093G)	6.11	123.40	41.94 protein unstable	0.690	H11 = 3.17 alpha helix	transmembrane section
T164A (A3796G)	6.11	123.40	41.94 protein unstable	0.690	H7 = 0.01 alpha helix	transmembrane section
Normal protein	6.11	123.08	41.94 protein unstable	0.682	H5 = 0.07<br>H7 = 0.01<br>H10 = 0.70<br>H11 = 1.95<br>H13 = 8.32	
ND2						
A331T (G5460A)	9.84	118.93	34.35 protein stable	0.629	H20 = 0.01 310 helix	transmembrane section, low complexity area
V193I (G5046A)	9.84	119.51	34.90 protein stable	0.637	H11 = 0.65 alpha helix	transmembrane section
Normal protein	9.84	119.22	34.35 protein stable	0.636	H11 = 0.66<br>H20 = 0.37	
ND3						
T114A (A10398G)	4.33	140.87	48.95 protein unstable	1.014	beta roll	transmembrane section
I9T (T10084C)	4.33	136.61	49.07 protein unstable	0.947	H1 = 40.47 alpha helix	transmembrane section, low complexity area
Normal protein	4.33	140.00	50.62 protein unstable	0.992	H1 = 53.87	
ND4						
I165T (T11253C)	9.40	127.73	35.98 protein stable	0.716	H8 = 1.16 alpha helix	transmembrane section
Normal protein	9.40	128.58	35.98 protein stable	0.727	H8 = 2.56	

			ND5			
S270N (G13145A)	9.14	116.12	33.37 protein stable	0.567	H15 = 1.19 alpha helix	transmembrane section
Y159H (T12811C)	9.15	116.12	34.15 protein stable	0,568	beta roll	transmembrane section
V24I (G12406A)	9.14	116.29	34.15 protein stable	0.572	H2 = 0.02 alpha helix	mitochondrial matrix
C518Y (G13889A)	9.18	116.12	33.47 białko stabilne	0.565	H24 = 0.02 alpha helix	mitochondrial matrix
T556I (C14003T)	9.14	116.77	34.40 protein stable	0.580	H26 = 0.02 pi helix	mitochondrial matrix, low complexity area
N471S (A13748G)	9.14	116.12	33.42 protein stable	0.576	H22 = 0.49 alpha helix	transmembrane section
S423T (G13604C)	9.14	116.12	34.01 protein stable	0.572	H21 = 1.13 alpha helix	transmembrane section
A475T (G13759A)	9.14	115.96	33.83 protein stable	0.567	beta roll	transmembrane section
Normal protein	9.14	116.12	34.15 protein stable	0.572	H2 = 0.02 H15 = 1.23 H21 = 0.61 H22 = 0.48 H24 = 0.02 H26 = 0.03	
			ND6			
V31A (A14582G)	4.18	124.66	29.48 protein stable	1.057	H2 = 0.37 alpha helix	transmembrane section, low complexity area
Normal protein	4.18	125.75	29.48 protein stable	1.071	H2 = 0.21	
			Mutations			
			ND5			
V254M (G13096A)	9.14	115.64	34.35 protein stable	0.568	H13 = 0.42 alpha helix	transmembrane section
Y506STP (C13854A)	8.52	114.35	32.87 protein stable	0.603	H23 = 1.23 alpha helix	transmembrane section
Normal protein	9.14	116.12	34.15 protein stable	0.572	H13 = 0.42 H23 = 2.52	

Abbreviations: ND1, ND2, ND3, ND4, ND4L, ND5, ND6 – subunits of NADH dehydrogenase - complex I of the respiratory chain; H - histidine; Y - tyrosine; A - alanine; T - threonine; M - methionine; I - isoleucine;V - valine; N - asparagine; S - serine; C – cysteine.

Table S2. The frequency of occurrence of the amino acid residue depending on the position in the protein, assessed using the PSSM viewer program for complex I (ND1, ND2, ND3,ND4, ND5, ND6).

**Polymorphisms**			
ND1 (MTH00104)			
The rest of the amino acid	F_r	F_w	PSSM score
Y304H			
H	0.69	0.54	9
Y	0.24	0.32	6
T67A			
A	0.00	0.01	0
T	0.14	0.21	3
T229M			
M	0.11	0.17	4
T	0.25	0.27	4
T240A			
A	0.01	0.03	−1
T	0.27	0.32	4
T263A			
A	0.08	0.07	1
T	0.74	0.77	6
T164A			
A	0.05	0.08	0
T	0.77	0.63	5
ND2 (MTH00105)			
The rest of the amino acid	F_r	F_w	PSSM score
A331T			
T	0.10	0.13	2
A	0.01	0.01	−3
V193I			
I	0.68	0.69	7
V	0.26	0.22	3
ND3 (MTH00106)			
The rest of the amino acid	F_r	F_w	PSSM score
T114A			
A	0.18	0.15	1
T	0.60	0.55	5
I9T			
T	0.62	0.53	5
I	0.32	0.39	5
ND4 (MTH00110)			
The rest of the amino acid	F_r	F_w	PSSM score
I165T			
T	0.10	0.11	1
I	0.30	0.33	4
ND5 (MTH00108)			
The rest of the amino acid	F_r	F_w	PSSM score
S270N			
N	0.95	0.95	8
S	0.04	0.04	0
Y159H			
H	0.24	0.29	7
Y	0.63	0.60	8
V24I			
I	0.14	0.18	3
V	0.03	0.05	0
C518Y			
Y	0.04	0.06	3
C	0.00	0.00	−1
T556I			

I	0.48	0.39	5
T	0.27	0.28	4
	N471S		
S	0.22	0.21	3
N	0.72	0.70	7
	S423T		
T	-	-	-
S	1.00	1.00	7
	A475T		
T	0.59	0.56	6
A	0.02	0.03	−1
	ND6 (MTH00109)		
The rest of the amino acid	F_r	F_w	PSSM score
	V31A		
A	-	-	-
V	0.08	0.15	2
	Mutations		
	ND5 (MTH00108)		
The rest of the amino acid	F_r	F_w	PSSM score
	V254M		
M	0.01	0.02	0
V	0.98	0.97	7

Bold type indicates an amino acid that appears in the reference sequence in mtDB - Human Mitochondrial Genome Database (http://www.mtdb.igp.uu.se/; Uppsala, Sweden). Normal font indicates the amino acid in the test material. Explanations in the list of abbreviations. Abbreviations: ND1, ND2, ND3, ND4, ND4L, ND5, ND6 – subunits of NADH dehydrogenase - complex I of the respiratory chain; Fr (raw frequency/ unweighted frequency) – initial frequency - these are the initial frequency bars showing the real (unweighted) residual frequencies at each position of the seed alignment in conserved domains (CD). The characters in the gaps are treated as normal amino acids in these calculations; Fw (weighted frequency) - the frequency weighted column shows the calculated frequencies using the procedure Henikoff, JG. J Mol Biol. 1994; 243:574-578, modified as described on page 3395 of Altschul SF, et al. Nucleic Acids Res. 1997; 25: 3389-3402. Briefly, sequences similar in seed alignment have less weight in calculating the frequency of the residues as these sequences provide redundant information; H - histidine; Y - tyrosine; A - alanine; T - threonine; M - methionine; I - isoleucine;V - valine; N - asparagine; S - serine; C – cysteine.

Table S3. Evaluation of the conservativeness and the effect of changes in amino acid residues occurring in the subunit of complex I on protein function using the SIFT Sequence and ConSurf Database programs.

Change in protein position	Nucleotide sequence change	The frequency of occurrence of the reference sequence from mtDB – Human Mitochondrial Genome Database	The frequency of occurrence of the change sequence from mtDB – Human Mitochondrial Genome Database	Score value based on evaluation of the effect on protein function in SIFT Sequence (harmfulness of the change)	Conservativeness - normalized scores	Conservativeness - on a scale of 1–9
Polymorphisms						
ND1						
Y304H	T4216C	2460	244	1.00 (tolerated)	0.572	3
T67A	A3505G	2648	56	0.02 (affects the functioning of the protein)	−0.711	7
T229M	C3992T	2680	24	0.05 (affects the functioning of the protein)	0.464	3
T240A	A4024G	2682	22	0.03 (affects the functioning of the protein)	0.202	4
T263A	A4093G	2702	2	0.38 (tolerated)	0.457	4
T164A	A3796G	2677	10	0.64 (tolerated)	1.769	1
ND2						
A331T	G5460A	2528	176	0.41 (tolerated)	0.702	3
V193I	G5046A	2625	79	1.00 (tolerated)	−0.149	5
ND3						
T114A	A10398G	1461	1242	0.66 (tolerated)	0.167	4
I9T	T10084C	2673	31	0.39 (tolerated)	0.749	3
ND4						
I165T	T11253C	2694	10	0.11 (tolerated)	−0.197	6
ND5						
S270N	G13145A	2687	16	1.00 (tolerated)	0.149	5
Y159H	T12811C	2667	37	0.63 (tolerated)	−0.336	6
V24I	G12406A	2649	55	060 (tolerated)	0.070	5
C518Y	G13889A	2697	7	1.00 (tolerated)	2.400	1
T556I	C14003T	2702	2	0.50 (tolerated)	−0.196	6
N471S	A13748G	2702	2	0.51 (tolerated)	0.516	3
S423T	G13604C	-	-	0.00 (affects the functioning of the protein)	−0.908	8

A475T	G13759A	2665	39	0.77 (tolerated)	0.684	3
ND6						
V31A	A14582G	2681	23	0.96 (tolerowana)	-0.678	8
Mutations						
ND5						
V254M	G13096A	-	-	0.04 (affecting the function of the protein)	−1.252	9

Scoring according to SIFT Sequence: change tolerated > 0.05, change affects protein function ⩽ 0.05.

Conservativeness on a scale of 1 - 9: 1 - 3 variable region, 4 - 6 region of medium conservativeness, 7 - 9 region highly conserved. Conservativeness by normalized score: variable region < 0, region moderately conserved 0 - 0.5, highly conserved region > 0.5. Abbreviations: H - histidine; Y - tyrosine; A - alanine; T - threonine; M - methionine; I - isoleucine;V - valine; N - asparagine; S - serine; C – cysteine; ND1, ND2, ND3, ND4, ND4L, ND5, ND6 – subunits of NADH dehydrogenase - complex I of the respiratory chain.

Table S4. Assessment of the pathogenicity of amino acid residue changes occurring in the subunit of complex I in the MitImpact3D program (APOGEE predictor).

Change in protein position	Nucleotide sequence change	Pathogenicity (scoring according to APOGEE)
	Polymorphisms	
	ND1	
Y304H	T4216C	Pathogenic (0.73)
T67A	A3505G	Neutral (0.31)
T229M	C3992T	Neutral (0.38)
T240A	A4024G	Neutral (0.38)
T263A	A4093G	Neutral (0.36)
T164A	A3796G	Neutral (0.38)
	ND2	
A331T	G5460A	Neutral (0.33)
V193I	G5046A	Neutral (0.31)
	ND3	
T114A	A10398G	Neutral (0.44)
I9T	T10084C	Neutral (0.37)
	ND4	
I165T	T11253C	Pathogenic (0.53)
	ND5	
S270N	G13145A	Neutral (0.29)
Y159H	T12811C	Neutral (0.32)
V24I	G12406A	Pathogenic (0.51)
C518Y	G13889A	Neutral (0.29)
T556I	C14003T	Neutral (0.41)
N471S	A13748G	Neutral (0.33)
S423T	G13604C	Neutral (0.41)
A475T	G13759A	Neutral (0.4)
	ND6	
V31A	A14582G	Neutral (0.26)
	Mutations	
	ND5	
V254M	G13096A	Neutral (0.31)

APOGEE scoring: neutral change ⩽ 0.5, pathogenic change> 0.5. Abbreviations: H - histidine; Y - tyrosine; A - alanine; T - threonine; M - methionine; I - isoleucine;V - valine; N - asparagine; S - serine; C – cysteine; ND1, ND2, ND3, ND4, ND4L, ND5, ND6 – subunits of NADH dehydrogenase - complex I of the respiratory chain.
